# Augmenting the Activity of Chlorhexidine for Decolonization of *Candida auris* from Porcine skin

**DOI:** 10.3390/jof7100804

**Published:** 2021-09-25

**Authors:** Chad J. Johnson, Emily F. Eix, Brandon C. Lam, Kayla M. Wartman, Jennifer J. Meudt, Dhanansayan Shanmuganayagam, Jeniel E. Nett

**Affiliations:** 1 Department of Medicine, University of Wisconsin, Madison, WI 53706, USA; cjohnson@medicine.wisc.edu (C.J.J.); eix@wisc.edu (E.F.E.); bclam@wisc.edu (B.C.L.); kmwartman@wisc.edu (K.M.W.); 2 Department of Medical Microbiology and Immunology, University of Wisconsin, Madison, WI 53706, USA; 3 Department of Animal and Dairy Sciences, University of Wisconsin, Madison, WI 53706, USA; jmeudt@wisc.edu (J.J.M.); dshanmug@wisc.edu (D.S.); 4 Department of Surgery, University of Wisconsin School of Medicine and Public Health, Madison, WI 53726, USA; 5 Center for Biomedical Swine Research and Innovation, University of Wisconsin School of Medicine and Public Health, Madison, WI 53726, USA

**Keywords:** *Candida auris*, biofilm, chlorhexidine, skin, porcine, essential oil, decolonization, isopropanol, *Melaleuca*, Cymbopogon

## Abstract

*Candida auris* readily colonizes skin and efficiently spreads among patients in healthcare settings worldwide. Given the capacity of this drug-resistant fungal pathogen to cause invasive disease with high mortality, hospitals frequently employ chlorhexidine bathing to reduce skin colonization. Using an ex vivo skin model, we show only a mild reduction in *C. auris* following chlorhexidine application. This finding helps explain why chlorhexidine bathing may have failures clinically, despite potent in vitro activity. We further show that isopropanol augments the activity of chlorhexidine against *C. auris* on skin. Additionally, we find both tea tree (*Melaleuca alternifolia*) oil and lemongrass (*Cymbopogon flexuosus*) oil to further enhance the activity of chlorhexidine/isopropanol for decolonization. We link this antifungal activity to individual oil components and show how some of these components act synergistically with chlorhexidine/isopropanol. Together, the studies provide strategies to improve *C. auris* skin decolonization through the incorporation of commonly used topical compounds.

## 1. Introduction

Over the past decade, *Candida auris* has appeared as a major cause of nosocomial invasive fungal infection [1]. In areas where it first emerged, *C. auris* now accounts for up to nearly 20% of candidemias [2,3]. *C. auris* has been termed a global public health threat based on its ability to spread rapidly in healthcare settings and cause invasive disease with a mortality rate approaching 60% [1,4,5,6]. Compared to other *Candida* spp., such as *C. albicans*, *C. auris* exhibits a high capacity for skin colonization, a characteristic that likely contributes to patient-to-patient transmission [5,7,8]. 

*C. auris* skin colonization places patients at risk for invasive disease. Invasive candidiasis often occurs in patients with indwelling medical devices, such as vascular catheters and G-tubes, which appear to serve as portals for fungal entry [1,9]. Sites of reported colonization include the axilla, groin, and nares, and patients can remain colonized for months [7]. In attempts to decolonize the skin, healthcare providers often use antiseptics for cleansing patients. One of the most commonly utilized agents is chlorhexidine, a broad-spectrum biocide with excellent in vitro activity against *C. auris* [10,11]. However, it is notable that hospital transmission often persists despite the use of chlorhexidine for decolonization, even with the implementation of other targeted infection control measures [12]. In these settings, *C. auris* has been reported to persist on the skin of patients despite chlorhexidine decolonization attempts [5,12,13]. In addition, *C. auris* has readily spread in healthcare institutions that routinely perform chlorhexidine bathing on all patients [14]. 

Here, we examine the activity of chlorhexidine against *C. auris* during the colonization of porcine skin using an ex vivo model [8]. Porcine skin was selected based on the many characteristics that it shares with human skin, including skin thickness, repair mechanisms, and cell types [15,16,17,18]. We find that, despite the potency of chlorhexidine in vitro, the antiseptic does not eradicate *C. auris* from porcine skin. However, the activity of chlorhexidine could be augmented with the addition of isopropanol and the active components of two essential oils. 

## 2. Materials and Methods

### 2.1. Organisms and Inoculum 

All studies included the *C. auris* clinical isolate B11203 provided by the Centers for Disease Control and Prevention (CDC) [1]. For comparison studies shown in Table S2, additional clinical *C. auris* isolates provided by the CDC (B11220, B11221, and B11801) were included [1]. Strains were maintained on yeast extract–peptone–dextrose (YPD) plates (1% Bacto^TM^ yeast extract, 2% Bacto^TM^ peptone, 2% Bacto^TM^ agar (Bacto^TM^ products from Becton, Dickinson and Company, Sparks, MD, USA) and 2% dextrose (Neogen Corp., Lansing, MI, USA) in water, and propagated in YPD broth (1% Bacto^TM^ yeast extract, 2% Bacto^TM^ peptone and 2% Dextrose)) overnight in an orbital shaker at 30 °C. Overnight cultures were diluted 1:1000 in Dulbecco’s phosphate-buffered saline (DPBS) (Lonza, Walkersville, MD, USA), enumerated by hemocytometer, and adjusted to 10^7^ cells/mL in synthetic sweat media. The synthetic sweat media was prepared as previously described [8].

### 2.2. Reagents 

Our experiments studied topical antiseptics commonly used in healthcare settings and essential oils with antimicrobial activity, including chlorhexidine gluconate (Frontier Scientific, Logan, UT, USA), chlorhexidine-isopropanol (ChloraPrep^®^ One-Step, CareFusion, El Paso, TX, USA), isopropanol (Fisher Scientific, Fairlawn, NJ, USA), antiseptic soap (Bright solutions, Chattanooga, TN, USA), tea tree (*Melaleuca alternifolia*) oil (MilliporeSigma, St. Louis, MO, USA), and lemongrass (*Cymbopogon flexuosus*) oil (NOW Foods, Bloomingdale, IL, USA). For in vivo experiments, antiseptics were applied at the recommended concentrations. In vitro experiments included the same recommended concentrations or series of dilutions. In vitro experiments also included the evaluation the major active components of the essential oils (citral, linalool, terpinen-4-ol, α-terpinene, γ-terpinene, 3-carene and eucalyptol, MilliporeSigma, St. Louis, MO, USA). 

### 2.3. Porcine Skin Model 

The collection of porcine skin samples was conducted under protocols approved by the University of Wisconsin–Madison Institutional Animal Care and Use Committee in accordance with published National Institutes of Health (NIH) and United States Department of Agriculture (USDA) guidelines. Biopsy samples were obtained from the excised skin of euthanized animals, as previously described [8]. Full-thickness skin samples were placed into the wells of 12-well plates containing 3 mL Dulbecco’s Modified Eagle Medium (DMEM) (Lonza, Walkersville, MD, USA), supplemented 10% FBS (Atlanta biologicals, Lawrenceville, GA, USA), penicillin (1000 U/mL), and streptomycin (1 mg/mL) (Corning, Manassas, VA, USA) for 6 h. Tissues were then rinsed in DPBS and placed on semi-solid media (6:4 ratio of 1% agarose (BIO-RAD, Hercules, CA, USA) in DPBS:DMEM with 10% FBS) and surrounded by paraffin wax. To colonize the skin, *C. auris* suspended in synthetic sweat media (10^7^ cells/mL) was applied to the skin surface (10 µL), and the samples were incubated for 24 h without a humidity source at 37 °C with 5% CO_2_. To assess the activity of topical treatments, reagents were applied for 1 h at 37 °C and then removed with a sterile swab. Following removal, 10 µL synthetic sweat media was re-applied, and skin samples were incubated for 24 h as described above, repeating for a total of 72 h (3 applications). Samples were then vortexed in DPBS and plated for assessment of viable burden or processed for histopathology (described below).

### 2.4. Histopathology 

Skin samples were rinsed in DPBS and then placed in 10% buffered formalin for 48 h and embedded in paraffin. Sections (10 µm) were stained with modified Grocott’s methenamine silver stain kit (Richard-Allan Scientific, Kalamazoo, MI, USA) according to the manufacturer’s instructions. Sections were imaged on an Echo Rebel (Echo, San Diego, CA, USA) at 40X. 

### 2.5. In Vitro Assessment of Antimicrobial Activity 

To evaluate the activity of antiseptics in vitro, *C. auris* was propagated as biofilm in synthetic sweat media, as previously described [8]. Briefly, *C. auris* (10 µL of 10^7^ yeast/mL) was added to flat-bottom 12-well microtiter plates and the plates were incubated for 24 h at 37 °C with 5% CO_2_ [8]. Antiseptics (50 µL) were gently added over biofilm, taking care not to disturb the architecture, for 1 h at 37 °C. Biofilms were then recovered by adding 440 μL of DPBS, dislodging with pipette tips, and then moving the samples to 1.5 mL tubes. An additional 500 µL DPBS was added to the plate and pipetted to recover any remaining yeast. Samples were then vortexed and centrifuged at 1200× *g* for 5 min, and the supernatant was removed afterward. Yeast pellets were resuspended by vortexing in 100µL DPBS. Samples (10 µL) were then plated in triplicate for the assessment of viable burden (or diluted and then plated). The concentrations used for analysis of individual oil components are shown in Supplementary Table S1. 

### 2.6. Biofilm Minimal Inhibitory Concentrations Testing (BMIC) 

To assess the potency of the antiseptics and oil components over a range of concentrations, we adapted the assay for Biofilm Minimal Inhibitory Concentrations Testing (BMIC). In these assays, biofilms were similarly propagated in 96-well plates. Biofilms were subsequently treated with 100 µL of serial dilution compounds for 1 h at 37 °C. The agents were then removed and 150 µL of RPMI-MOPS (10.4 g RPMI medium powder with L-glutamine and without sodium bicarbonate (Life Technologies, Grand Island, NY, USA) and 34.5 g MOPS (Fisher Scientific, Fair Lawn, NJ, USA) dissolved in ddH_2_O, with its pH brought to 7 with sodium hydroxide and its total volume brought to 1 L with ddH_2_O, and filter sterilization) was added for 24 h. Overnight growth was assessed by OD_600_ values obtained using a microplate reader (Synergy H1, Bio-Tek Instruments) [19]. BMIC was defined as the lowest concentration that resulted in at least 50% inhibition compared to controls. 

### 2.7. Antiseptic Combinations Studies 

For studies analyzing the interactions between compounds, checkerboard assays were employed to calculate fractional inhibitory concentration indices (FICI) [20]. *C. auris* biofilms were formed in 96-well flat-bottom plates, as described above, and treated with 100 µL dilutions of combinations of chlorhexidine and essential oils or component dilutions in a checkerboard format for 1 h at 37 °C. Antiseptics were then removed and 150 µL RPMI-MOPS was added. After 24 h at 37 °C, OD600 was determined in the plate reader and BMIC^comb^ measurements were determined. The FIC index was expressed as ΣFIC = FICA + FICB = BMIC^comb^/BMIC^alone^ + BMIC^comb^/BMIC^alone^_._ The interactions were identified as synergistic if ΣFIC ≤ 0.50, additive or indifferent if ΣFIC ranged from >0.50 to ≤4.0, and antagonistic if ΣFIC > 4.0.

## 3. Results

### 3.1. C. auris Persists on Porcine Skin following the Application of Hospital Antiseptic Cleansers

**Figure 1:**
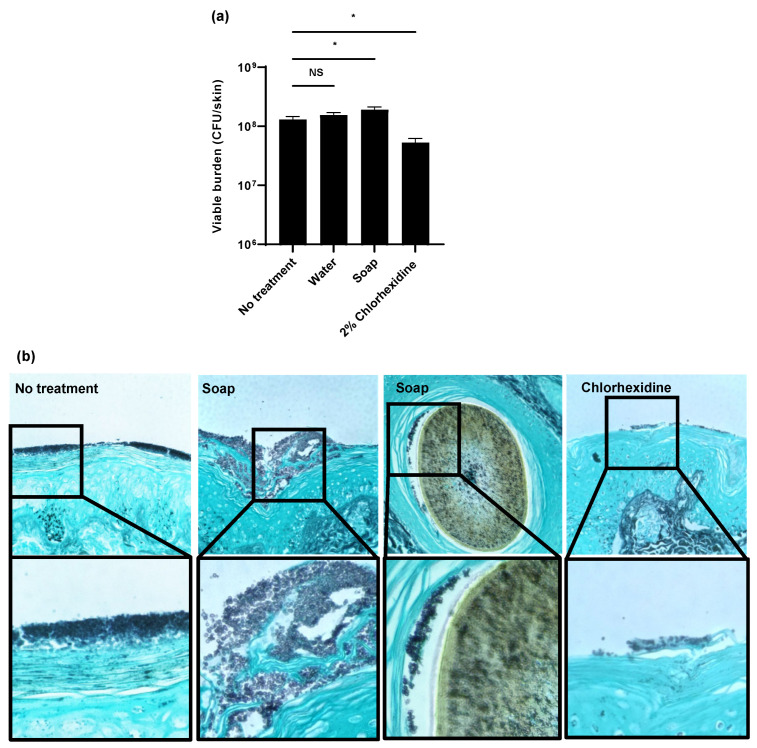
Impact of biocides on *C. auris* colonization of porcine skin: (**a**,**b**) *C. auris* was applied to the surface of full-thickness porcine skin samples and samples were incubated for 24 h to allow for colonization. Water, soap (with 0.3% chloroxylenol), or 2% chlorhexidine gluconate were applied for 1 h daily for 3 days. The impact on colonization was assessed by viable burden (**a**) and histopathology with Grocott’s methenamine silver staining (**b**). With this stain, fungal elements appear dark purple and the hair is brown. Data were analyzed by one-way ANOVA with Holm–Sidak multiple comparison to the control, * *p* < 0.05, NS = not significant, standard error of the mean shown, *n* = 3.

To evaluate the efficacy of hospital skin cleansers on *C. auris* skin colonization, we utilized an ex vivo pig skin model [8]. For these studies, we selected a miniature pig model (Wisconsin Miniature Swine^TM^), which closely mimics human skin composition [8,15,21]. To colonize the skin, we applied *C. auris* suspended in synthetic sweat media. Following a 24 h incubation, we applied several antiseptic washes for 1 h daily for 3 days to mimic the bathing of patients in a hospital setting. We first examined a water-only control and an antiseptic soap typically used for hand washing in hospitals and in homes (0.3% chloroxylenol) [22]. We also included chlorhexidine, an antiseptic often utilized to decolonize *C. auris* from the skin of patients and for the routine bathing of patients [5,13,22]. Treatment with water only or the antiseptic soap did not decrease the burden of C. auris when compared to the no treatment controls (Figure 1a). Surprisingly, the treatment with the antiseptic soap resulted in a slightly higher burden of *C. auris* skin colonization. We did observe a drop in fungal burden upon treatment with 2% chlorhexidine (Figure 1a). However, this 0.5 log-reduction was lower than expected given the potency (several log-reduction) of this agent against *C. auris* in vitro [10,11]. 

As a second method to assess skin colonization, we utilized histopathology with Grocott’s methenamine silver (GMS) staining (Figure 1b). Without treatment, we observed *C. auris* (stained dark purple) to form thick multilayers of yeast adhering to the epidermal surface. Treatment with antiseptic soap did not appear to decrease the fungal burden. Consistent with the viable burden studies, we actually observed more yeast adhering to the skin of soap-treated samples when compared to the no-treatment controls. In these samples, we identified *C. auris* (dark purple) adjacent to hair (brown) within the hair follicles. As this was not observed in the other samples, application of the antiseptic soap appeared to promote *C. auris* growth in the hair follicles. In contrast, treatment with chlorhexidine visibly decreased the total surface-associated fungal burden. However, yeast remained in the most superficial epidermal layers.

### 3.2. Isopropanol Augments the Activity of Chlorhexidine for C. auris Skin Decolonization

**Figure 2:**
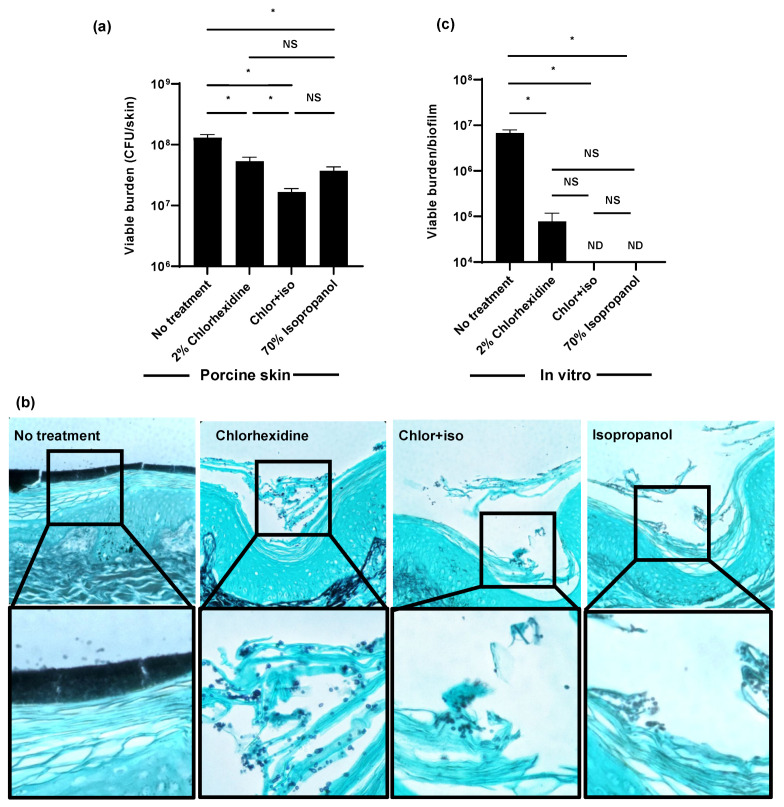
Isopropanol augments the activity of chlorhexidine for *C. auris* skin decolonization: (**a**,**b**) *C. auris* was applied to the surface of full-thickness porcine skin samples and samples were incubated for 24 h to promote colonization. Chlorhexidine gluconate (2%), chlor+iso (2% chlorhexidine + 70% isopropanol), or isopropanol (70%) was applied for 1 h daily for 3 days. The impact on colonization was assessed by viable burden (**a**) and histopathology with Grocott’s methenamine silver staining (**b**). (**c**) *C. auris* biofilms were propagated in synthetic sweat media in 12-well plates for 24 h. Antiseptics were applied for 1 h and the remaining viable burden was determined by plating. ND = not detected (CFU < 10 colonies). Data were analyzed by one-way ANOVA with Holm–Sidak multiple comparison among groups, * *p* < 0.05, NS = not significant, standard error of the mean shown, *n* = 3.

In a hospital setting, isopropanol is often co-administered with chlorhexidine to enhance its activity. Skin cleansing with this combination is typically performed in pre-operative settings for the prevention of surgical wound infection, and prior to the placement of vascular catheters for the prevention of catheter-associated bloodstream infection [23,24]. Using the ex vivo porcine skin model, we found the preparation of 2% chlorhexidine with 70% isopropanol (chlor+iso) decreased *C. auris* colonization by 1 log-reduction (Figure 2a). The activity of chlor+iso was greater than the activity of either component administered separately. As a second method to assess skin colonization, we utilized histopathology with Grocott’s methenamine silver staining for fungal elements (Figure 2b). Without treatment, *C. auris* densely colonized the epidermal surface. Consistent with viable burden data, decreased *C. auris* colonization was observed with chlorhexidine, chlor+iso, and isopropanol treatment. Compared to the chlorhexidine-treated skin sample, chlor+iso treatment appeared to further decrease fungal abundance. For each of the treated samples, *C. auris* appeared to persist in the superficial epidermal layers while the surface-adherent multilayer structure was no longer present.

Given the minimal activity of chlorhexidine on *C. auris* skin colonization ex vivo, we considered that our clinical isolate may exhibit a resistance to chlorhexidine different from that reported in the literature [10,11]. For these experiments, we grew a similar burden of *C. auris* in synthetic sweat for 24 h, applied antiseptic for 1 h, disrupted the biofilms, and determined the viable burden by plating (Figure 2c). We found single administration of chlorhexidine decreased the fungal burden by an over 2 log-reduction. Both chlor+iso and isopropanol exhibited potent in vitro activity, resulting in no detectable viable burden. These data suggested to us that the activity of chlorhexidine, chlor+iso, and isopropanol observed in vitro may not represent the action of these agents against *C. auris* on skin [25]. 

### 3.3. Tea Tree Oil (TTO) and Lemongrass Oil (LGO) Enhance the Activity of Chlor+Iso for C. auris Decolonization of Skin

**Figure 3:**
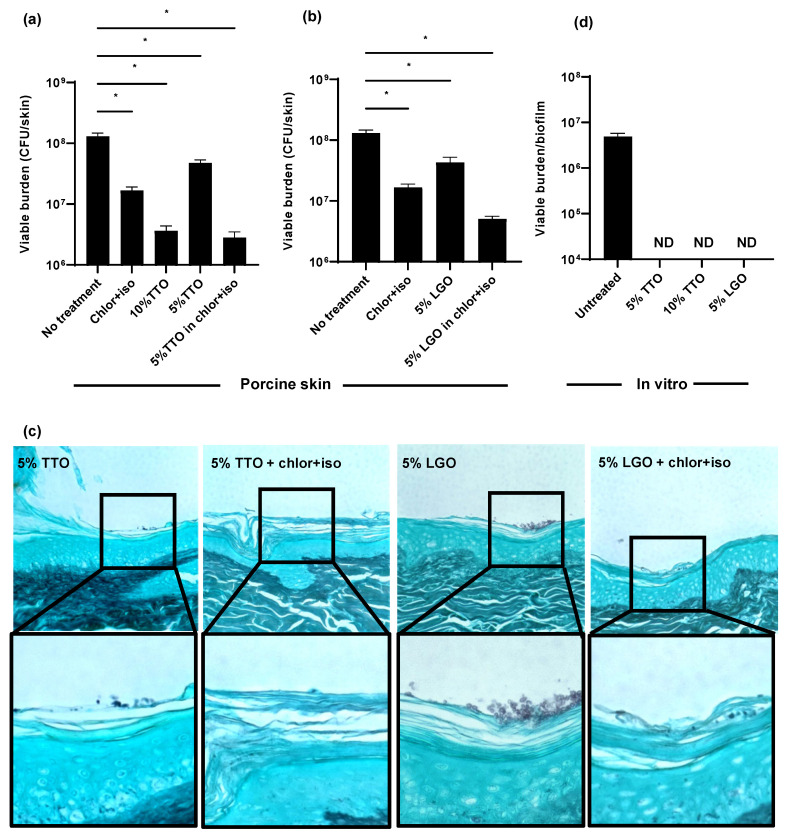
Impact of biocides on *C. auris* skin colonization and in vitro: (**a**–**c**) *C. auris* was applied to the surface of full-thickness porcine skin samples. After 24 h, biocides were applied for 1 h, and samples were incubated for 24 h. The impact on colonization was assessed by viable burden (**a**,**b**) and histopathology with Grocott’s methenamine silver staining (**c**). (**d**) *C. auris* biofilms were propagated in synthetic sweat media in 12-well plates for 24 h. Essential oils were applied for 1 h and the remaining viable burden was determined by plating. Data were analyzed by one-way ANOVA with Holm–Sidak multiple comparison on the control, ND = not detectable, * *p* < 0.05, NS = not significant, standard error of the mean shown, *n* = 3.

Although the addition of isopropanol to chlorhexidine improved activity, treatment with this combination (chlor+iso) for 3 days still only lowered the *C. auris* skin colonization burden by 1 log-reduction (Figure 3a). The discrepancy between the potent activity of chlor+iso in vitro and the lower activity on skin led us to question whether its delivery to *C. auris* propagating within the epithelial skin layers may be limited. We considered the possibility that essential oils with antifungal activity may be helpful for decolonization. Essential oils from a variety of plants have been shown to exhibit broad antifungal effects and have been utilized for topical use [26]. The active components of these oils consist primarily of terpenes and their metabolic derivatives, and vary by derived plant genus and species. We elected to examine the activity of tea tree (Melaleuca alternifolia) oil, given its antifungal effects and documented favorable safety profile [25,26,27]. We also included lemongrass (Cymbopogon flexuosus) oil, in light of its antifungal properties and worldwide use [28]. Concentrations were selected based on reported safety profiles [25,29]. 

Using the porcine skin model, we examined the efficacy of TTO and LGO on *C. auris* colonization alone and in combination with chlor+iso. Treatment with 5% TTO in combination with chlor+iso lowered the *C. auris* skin colonization burden by 1.5 log-reduction CFUs, a greater reduction than either chlor+iso or 5% TTO alone (Figure 3a). When applied alone, we found that 10% TTO similarly reduced fungal burden by 1.5 log-reduction. Likewise, treatment with 5% LGO in combination with chlor+iso resulted in a greater reduction in fungal colonization than either chlor+iso or 5% LGO alone (Figure 3b). To further assess the impact of these essential oils on skin colonization, we used histopathology with GMS staining (Figure 3c). In line with our fungal burden results, we observed lower skin colonization for combined treatments with essential oils and chlor+iso compared to samples treated with 5% TTO or LGO alone. These data suggest that both TTO and LGO augment the activity of chlor+iso in reducing *C. auris* skin colonization burden. 

Given the differences observed for the in vitro and ex vivo activities of chlor+iso (Figure 2), we next investigated the in vitro efficacy of TTO and LGO against *C. auris*. After growing *C. auris* in synthetic sweat for 24 h, we applied TTO (5% and 10%) and LGO (5%) for 1 h, then determined viable burden by plating. We found that each of these treatments exhibited high activity against *C. auris* in vitro, with no detectable viable burden after treatment with either essential oil alone (Figure 3c). This suggests that, similar to other biocides, TTO and LGO exhibit potent in vitro activity against *C. auris* that is not fully recapitulated on skin. 

### 3.4. TTO and LGO and Their Active Components Are Synergistic with Chlorhexidine

**Figure 4:**
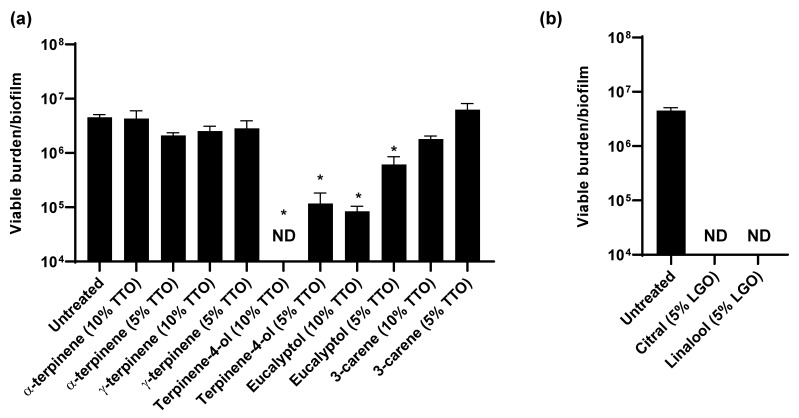
Impact of essential oil components on *C. auris* in vitro: (**a**,**b**) *C. auris* biofilms were propagated in synthetic sweat media in 12-well plates for 24 h. Essential oil components were applied for 1 h and the remaining viable burden was determined by plating. Component concentrations were selected based on the concentrations present in 5 or 10% TTO (**a**) or 5% LGO. Data were analyzed by one-way ANOVA with Holm–Sidak multiple comparison on the control, * *p* < 0.05, ND = not detectable, standard error of the mean shown, *n* = 3.

As essential oils contain a mixture of compounds, we next considered which of these components were exhibiting activity against *C. auris*. The primary components of TTO include α-terpinene, γ-terpinene, terpinen-4-ol, eucalyptol, and 3-carene [30]. To assess the individual activities of these, we calculated the concentrations present in 5% and 10% TTO and examined their impact on *C. auris* in vitro (Figure 4a). We found that treatment with terpinen-4-ol or eucalyptol significantly reduced the *C. auris* viable burden when compared to the untreated controls, indicating a role for these components in the activity of TTO against *C. auris* (Figure 4a). Terpinen-4-ol exhibited more potent activity, with the concentration present in 10% TTO eliminating viable growth. For the investigation of LGO, we examined citral and linalool, the major active components [30,31]. At concentrations present in 5% LGO, both of these components completely eliminated detectable viability, consistent with a role for both in the activity of LGO against *C. auris* colonization (Figure 4b).

Considering that TTO and LGO augment the activity of chlor+iso (Figure 3a,b), we questioned whether these interactions were additive or synergistic. For these analyses, we utilized checkerboard assays to calculate fractional inhibitory concentration indices (FICI) [20]. We adapted a biofilm minimum inhibitory concentration assay (BMIC) for calculations and examined the activities of serial dilutions of the essential oils and their components, alone and in combination with chlorhexidine (Table 1). Alone, LGO exhibited more potent activity than TTO, with BMICs of 0.0625% and 0.5%, respectively. Both displayed synergism with chlorhexidine. Of the essential oil components, citral was the most active with a BMIC of 0.03%. Citral also demonstrated synergistic activity with chlorhexidine, consistent with a role in LGO-chlor+iso synergism. For the TTO components, terpinen-4-ol exhibited more potent activity, while eucalyptol displayed synergism in combination with chlorhexidine.

*C. auris* isolates vary by geography and cluster into clades [1]. Strains have been shown to display different phenotypes with regard to drug resistance and virulence [1,32,33,34,35]. The isolate we selected for study was initially isolated from a patient in India and represents the Southeast Asian clade (clade I). To determine whether chlorhexidine, TTO, and LGO display similar activities against *C. auris* from other clades, we included isolates from clades II–IV in modified MIC assays. We observed similar responses among the strains. This suggests that the strategies to improve decolonization using these agents are likely to be applicable to a variety of *C. auris* strains (Table S2).

## 4. Discussion

*C. auris* readily colonizes human skin and spreads rapidly in hospitals, often in spite of infection control measures and skin decolonization efforts [5,12,13]. The development of more effective decolonization strategies is critical for controlling and eliminating this deadly pathogen. Here, we demonstrate that the common hospital disinfectant chlorhexidine only modestly reduces *C. auris* burden on porcine skin, despite its strong in vitro activity. We show that the activity of chlorhexidine is improved when combined with 70% isopropanol, and even further enhanced when combined with tea tree oil, lemongrass oil, or their active components. This suggests that current treatment strategies for *C. auris* decolonization could be improved by combining these well-tolerated antiseptics with chlorhexidine.

Recent studies utilize porcine skin as a model for *C. auris* colonization of human skin. While the skin of humans and pigs exhibit some differences, they have many similarities that have prompted the use of pig skin to model human skin ex vivo [15,16,17,18,21]. For example, the skins of both species have similar types of skin cells, thicknesses, vasculature, and skin repair mechanisms [15,16,17,18]. As many of these characteristics differ for rodents, pig skin is an ideal model for ex vivo work, particularly if human skin is not readily available.

In healthcare settings, *C. auris* can proliferate on patient skin despite daily washing with chlorhexidine [5,12,13]. One factor contributing to *Candida* persistence may be chlorhexidine’s limited skin penetration, as prior work with ex vivo human skin has shown the compound lacks permeation into deeper skin layers [36]. This poor penetration is anticipated to limit its effectiveness against microorganisms deeper in the skin, including hair follicles. In another work, Huang et al. found *C. auris* residing deep within the hair follicles of mice for months following initial colonization, consistent with an infectious reservoir [37]. Considering that chlorhexidine is expected to reach only minimal concentrations in the follicles, colonization at this site may help explain the difficulty of eliminating *C. auris* in healthcare settings.

Here, we show that treatment with combinations of chlorhexidine and isopropanol (chlor+iso) result in a greater reduction in *C. auris* both in vitro and on porcine skin. This finding is consistent with prior work demonstrating enhanced activity of this combination in vitro [38]. We further found that the essential oils TTO and LGO additionally enhanced the activity of chlor+iso on porcine skin. Multiple mechanisms may be contributing to the heightened activity for the combinations. For example, some components of essential oils readily permeate skin and can improve the penetration of various drugs, suggesting the possibility of improved chlorhexidine delivery [39,40]. In addition, we found synergistic interactions for combinations in vitro, consistent with the involvement of additional mechanisms separate from skin penetration. In bacterial studies, TTO components have been shown to compromise bacterial cytoplasmic membranes [41]. If the impact on *C. auris* is similar, membrane instability may increase susceptibility to chlorhexidine.

In healthcare settings, *C. auris* persists on surfaces and can be easily transmitted patient-to-patient, often by the use of contaminated, reusable medical devices [9,13,14]. However, transient hand colonization has been detected among healthcare workers and may contribute to outbreaks [13]. In a randomized study of hand hygiene, TTO was well tolerated and appeared to be effective for the prevention of healthcare-associated infections [42]. While further study in the area is needed, it is interesting to consider the addition of an essential oil and/or active component into hand washing regimens, particularly in areas of outbreaks.

## Figures and Tables

**Table 1:** Activities of essential oil components alone and in combination with chlorhexidine.

	Component % in 5% Oil (*v/v*)	Modified MIC (%)	FICI (Oil/Component + Chlorhexidine)	FICI Interpretation
**Tea tree oil**		0.5	0.375	Synergistic
terpinen-4-ol	1.9	0.25	0.625	Additive/indifferent
eucalyptol	0.7	2	0.22	Synergistic
**Lemongrass oil**		0.0625	0.376	Synergistic
citral	3.4	0.03	0.372	Synergistic
linalool	0.285	0.19	0.5	Additive/indifferent

## Data Availability

Not applicable.

## Supplementary Materials

The following are available online at https://www.mdpi.com/article/10.3390/jof7100804/s1, Table S1: Major oil components and associated toxicity; Table S2: Modified MICs for representative strains from Clades I-IV.
