# Supplementary material for: Augmenting the Activity of Chlorhexidine for Decolonization of Candida auris from Porcine skin
Source: J Fungi (Basel). 2021 Sep 25;7(10):804. doi: 10.3390/jof7100804 (PMC8537331; doi:10.3390/jof7100804)
Supplement: Supplementary file 1 [file jof-07-00804-s001.zip › jof-1364986-supplementary.pdf]

Supplementary Table S1. Major oil components and associated toxicity

	% of oil (v/v)	LD50	Ref
**Tea tree oil**			[1, 3-7]
terpinen-4-ol	38.2	>2500 mg/kg (skin - rabbit)	
3-carene	17	>2000 mg/kg (skin - rabbit)	
eucalyptol	13.9	>2000 mg/kg (skin - rat)	
α-terpinene	10.2	1680 mg/kg (oral - rat)	
γ-terpinene	2.0	3650 mg/kg (oral - rat)	
**Lemongrass oil**			[1, 2, 8-9]
citral	68.9	2250 mg/kg (skin - rabbit)	
linalool	5.7	5610 mg/kg (skin - rabbit)	

References

1. Krzysko-Lupicka T, Sokol S, Piekarska-Stachowiak AA. Evaluation of fungistatic activity of eight selected essential oils on four heterogeneous *Fusarium* isolates obtained from cereal grains in Southern Poland. Molecules 2020; 25.

2. Jackson GM, Hall DE, Walker R. Comparison of the short-term hepatic effects of orally administered citral in Long Evans hooded and Wistar albino rats. Food Chem Toxicol 1987; 25(505-13).

3. α-Terpinene. Food Chem Toxicol. 1976; 14(873).

4. γ-terpinene. Food Cosmet Toxicol. 1976; 14(875).

5. Terpinen-4-ol. Food Chem Toxicol. 1982; 20(833)

6. 3-carene. National Technical Information Service, OTS0533894.7. European Chemicals Agency (ECHA); Registered substances, Cineole (CAS Number: 470-82-6) (EC Number: 207-431-5) Food Chem Toxicol 1987; 25(505)

8. Linalool. Food Chem Toxicol. 1975; 13(827-32).

9. Gao S, Liu G, Li J, et al. Antimicrobial activity of lemongrass essential oil (*Cymbopogon flexuosus*) and its active component citral against dual-species biofilms of *Staphylococcus aureus* and *Candida* Species. Front Cell Infect Microbiol 2020; 10:603858.

Table S2. Modified MICs for representative strains from Clades I-IV

	Clade I B11203	Clade II B11220	Clade III B11221	Clade IV B11801
**Tea tree oil**	0.5%	0.125%	0.25%	0.25%
**Lemongrass oil**	0.062%	0.062%	0.062%	0.062%
**Chlorhexidine**	0.05%	0.012%	0.025%	0.025%
